# Comparison of total morphine milligram equivalents at hospital discharge between opioid-naive and opioid-experienced surgical patients: a single-centre retrospective cohort study

**DOI:** 10.1016/j.bjao.2025.100497

**Published:** 2025-10-17

**Authors:** Elis Liblik, Urs Pietsch, Anne-Katrin Hickmann

**Affiliations:** 1 Institute for Anaesthesiology and Perioperative Medicine, University Hospital Zurich, Zurich, Switzerland; 2 Department of Anaesthesiology Rescue and Pain Medicine and; 3 Division of Perioperative Intensive Care Medicine, Cantonal Hospital St Gallen, St Gallen, Switzerland; 4 Department of Emergency Medicine, Bern University Hospital (Inselspital), University of Bern, Bern, Switzerland; 5 Pain Clinic, Cantonal Hospital of St. Gallen, St. Gallen, Switzerland; 6 Neurochirurgisches Zentrum Ostschweiz, St. Gallen, Switzerland

**Keywords:** intravenous opioid, opioid dose at discharge, opioid status, patient-controlled analgesia, postoperative pain management, postsurgical opioid use

## Abstract

**Background:**

Perioperative pain management is a key concern amid the growing opioid pandemic, particularly for opioid-experienced patients. This retrospective single-centre cohort study aimed to compare morphine milligram equivalents (MME) at hospital discharge between opioid-naive and opioid-experienced adults undergoing surgery with postoperative patient-controlled analgesia (PCA). We hypothesised that opioid-experienced patients would require higher MME at discharge, and greater intraoperative remifentanil and postoperative PCA use.

**Methods:**

We retrospectively analysed 406 patients from 2016 to 2023 who received intravenous PCA for acute postoperative pain management. Trauma and neuraxial/regional block cases were excluded; emergency non-trauma cases included. Opioid-experienced patients were defined as chronic use of opioids for ≥3 months before surgery. The primary outcome was opioid dose at discharge in MME. Secondary outcomes were total intraoperative remifentanil dose and total PCA use in MME, analysed using multiple linear regression with permutation testing.

**Results:**

Opioid-experienced patients had a 15.4 MME day^−1^ higher discharge opioid dose (95% confidence interval [CI] 7.4–23.4 MME day^−1^; *P*<0.001), received 6.7× more opioids at discharge than opioid-naive patients (63.5 *vs* 9.4 MME day^−1^; *P*<0.001) and nearly doubled their own preoperative use (63.5 *vs* 30 MME day^−1^). Opioid-experienced patients also required 52.0 MME day^−1^ more via PCA (95% CI 13.1–90.8 MME day^−1^; *P*=0.009). Each additional preoperative MME was associated with a 0.9 MME day^−1^ increase in PCA use during the hospitalisation (95% CI 0.2–1.6 MME day^−1^; *P*=0.017).

**Conclusions:**

Preoperative opioid experience strongly predicted postoperative opioid requirements and discharge prescribing. Early identification of opioid-experienced patients and tailored multimodal strategies may improve individualised pain management. However, the retrospective single-centre design and lack of non-opioid analgesia data limit generalisability.

With almost every type of surgery, patients are administered opioids, which can increase the susceptibility to noxious stimuli and may bring about an opioid-induced hypersensitivity. Patients who are on chronic opioid therapy might develop a state of enhanced pain sensitisation (opioid-induced hyperalgesia).^1^, ^2^, ^3^ During the postoperative period, hyperalgesia leads to a higher opioid consumption and escalated pain sensibility. Opioid-induced hypersensitivity causes patient discomfort with higher pain scores, greater use of analgesics, and associated side effects.^4^, ^5^, ^6^ Therefore, the postsurgical pain management of opioid-experienced patients is particularly important.

Intravenous patient-controlled analgesia (PCA) is an effective and evidence-based approach for alleviating acute postoperative pain.^7^^,^^8^ Patients using PCA pumps have been proved to experience lower pain intensity scores and are generally more content with their pain management. However, they receive higher amounts of opioids than patients who are prescribed nurse-administered analgesia. Furthermore, Chung and colleagues^9^ recently showed that intraoperative remifentanil infusion remarkably increases the postoperative opioid consumption via intravenous fentanyl PCA, which could be attributed to opioid-induced hypersensitivity.^10^

High amounts of inpatient opioid use may bring about a rise in the demand for outpatient opioid prescriptions, which is why it is impending to find out how treating postoperative pain with PCA may influence opioid prescribing at discharge. The effect of a known daily preoperative opioid consumption should be taken into account as a variable, as the opioid tolerance and opioid-induced hypersensitivity may play a critical part in the need for postoperative analgesic requirements.

The primary aim of this study was to compare total morphine milligram equivalents (MME) prescribed at the time of hospital discharge between opioid-naive and opioid-experienced adult patients undergoing surgery with postoperative PCA at a single tertiary centre. Secondary aims were to evaluate the association of preoperative opioid use with intraoperative remifentanil dosage and postoperative PCA opioid requirements, expressed in total MME, during the acute postsurgical period. Opioid-experienced patients were defined as those with documented daily opioid use for at least three consecutive months before surgery, as recorded in the preoperative anaesthesia assessment. This group included both chronic non-cancer pain and cancer pain patients, without further subgroup differentiation.

We hypothesised that opioid-experienced patients would require higher MME at discharge compared with opioid-naive patients, and that they would also have greater intraoperative remifentanil and postoperative PCA opioid requirements.

## Methods

Data of all patients, who received a PCA pump for postsurgical analgesia at our institution between January 2016 and December 2023, were obtained from our prospectively maintained anaesthesia information management system (AIMS) database. Inclusion criteria were adult patients (≥18 yr) who underwent surgery under general anaesthesia with postoperative PCA. Patients with incomplete key data (*n*=16) were excluded before analysis, resulting in 781 patients eligible for screening. Trauma patients were excluded to better assess postsurgical pain management in a non-trauma context. Patients who received neuraxial anaesthesia, regional anaesthesia, or peripheral nerve blocks were also excluded to enable a direct evaluation of the relationship between preoperative opioid status and opioid requirements during surgery, in the acute postoperative phase with PCA, and at hospital discharge.

Opioid-experienced patients were defined as those with documented daily opioid use for at least three consecutive months before surgery, as recorded in the preoperative anaesthesia assessment. This group included both chronic non-cancer pain and cancer pain patients, without further subgroup differentiation. Patients who did not meet these criteria were classified as opioid naive.

All patients were treated according to our highly standardised institution’s protocol for intra- and postoperative pain management. Basis analgesia was managed with regular metamizole and paracetamol, with morphine as an option for acute pain management. The individual dosage of NSAIDs and adjuvant pain medication, such as neuropathic pain agents, was not assessed for this analysis with a focus on opioid needs. The general anaesthesia was carried out using target-controlled infusion (TCI) pumps based on the Schnider and Minto pharmacokinetic models. Intraoperative remifentanil dosage was adjusted based on clinical signs such as heart rate and blood pressure, as no analgesia monitoring systems (e.g. Analgesia Nociception Index [ANI], Surgical Plethysmographic Index [SPI]) were available. All anaesthesiologists followed standardised institutional protocols, ensuring consistent remifentanil titration across cases. The need for an opioid PCA pump was decided individually by pain consultants, based on patients’ previous medical data and type of surgery. In the recovery room a PCA pump with morphine 2 mg ml^−1^ or a PCA pump with fentanyl 0.05 mg ml^−1^ was installed.

The standard basic programming for morphine was bolus dose of 2 mg, bolus lockout for 8 min, without a basal rate, with a 4-h limit of 30 mg.

The standard basic programming for fentanyl was a bolus dose of 0.05 mg ml^−1^, lockout interval 10 min with a 4-h limit of 8 mL (= 400 μg).

Preoperative and postoperative opioid consumption were analysed by converting all opioid medications to MME for uniform analysis. The University Hospital Zurich Opimeter^11^ was used for this purpose, a specialised tool for calculating and converting opioid dosages. The exact length of pre-hospitalisation usage could not be extracted from the data, which is why this was not analysed.

The primary outcome was opioid dose at hospital discharge, expressed in MME. Secondary outcomes included intraoperative remifentanil dosage and postoperative opioid consumption via i.v. PCA (MME) during the acute postsurgical period.

The following variables were collected from patient charts and anaesthesia protocols for analysis: sex, age, ASA physical status classification, preoperative MME daily usage, intraoperative remifentanil dosage and duration of surgery, postoperative opioid dosage via PCA in MME during the acute postoperative phase, duration of the PCA pump use, and postoperative opioid daily usage at discharge in MME—this being the opioid prescription dosage at discharge.

Because of the considerable variability in subgroup sizes among the different surgical procedures, statistical analysis was not feasible.

**Fig 1:**
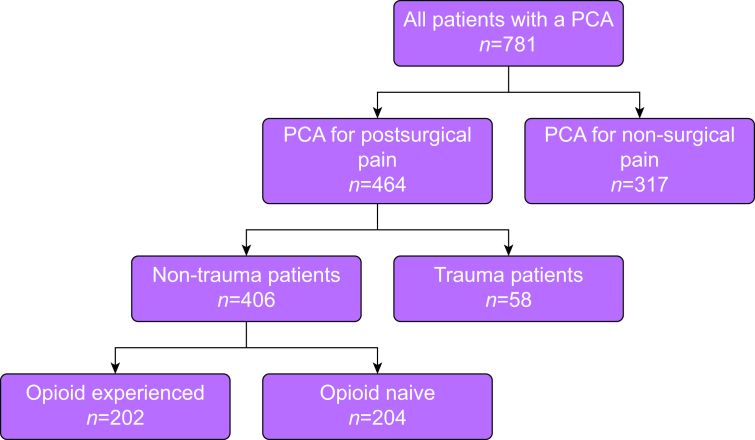
Flowchart outlining patient selection. PCA, patient-controlled analgesia.

This retrospective analysis was approved by the Ethics Committee of Eastern Switzerland (Project ID 2024-00153) and was performed and reported in accordance with the Strengthening the Reporting of Observational Studies in Epidemiology (STROBE) guidelines. A general consent for scientific use of routinely collected data was available for all patients; study-specific consent was not required. See Figure 1 for study enrolment flowchart.

### Statistical analysis

Cohort characteristics were analysed descriptively. For continuous variables, we reported the mean and standard deviation. The distributions of all continuous variables were assessed for normality using histograms and the Shapiro–Wilk test, within each group. When variables were not normally distributed, they were summarised using medians and inter-quartile ranges, and non-parametric tests were used for group comparisons

Data were compared between opioid-naive and opioid-experienced groups. Between-group comparisons included *P*-values and 95% confidence intervals (CI).

For power analysis simulation was used. We generated datasets for two groups with specified standard deviations and effect sizes, applied Welch’s *t*-test to each dataset, and repeated this process across numerous simulations. By calculating the proportion of tests where the null hypothesis was rejected, we estimated the test’s power. Based on the initial power analysis, a study sample of 200 was necessary to yield a power of 0.8, ensuring a high probability of detecting a true effect under the specified conditions.^–^12, 13, 14

Multiple linear regression with permutation testing to determine the significance of the model and its coefficients was performed. Permutation testing was to test partial regression coefficients in a linear model based on sample partial correlations. This approach allowed us to see if remifentanil was associated with the change in opioid dosage when administered via i.v. PCA in the presence of variables sex, age, ASA physical status classification, and preoperative opioid daily usage. We used the permutations test to determine variables in the model.

We assessed the cumulative consumption of postoperative opioids stratified by the length of the operation and in different age/sex/ASA physical status classification categories.

All statistical analyses were performed using IBM SPSS Statistics for Windows (version 26.0; IBM Corp, Armonk, NY, USA) and Python (including NumPy, SciPy, and Matplotlib libraries), as appropriate. Statistical significance was set at *P*<0.05.

## Results

The final study cohort consisted of 406 patients, comprising both planned elective procedures and emergency non-trauma surgeries (e.g. laparotomies), with a mean age of 52.4 (sd 17.7) yr. All patients had general anaesthesia after the TCI standard with propofol and remifentanil. The mean cumulative intraoperative remifentanil dosage was 1674.7 (1187.0) μg. The mean duration of surgery was 160.6 (133.6) min. 49.8% of patients had prior experience with opioid use. See Table 1 for the analysed patient characteristics and clinical variables.

**Table 1:** Patient characteristics and clinical variables. MME, morphine milligram equivalents; PCA, patient-controlled analgesia; μ, micrograms.

Variable, mean (sd)	Opioid Naive (*N*=204)	Opioid Experienced (*N*=202)	*P*-value
Age (yr)	53	52	0.565
Sex (proportion female)	0.00 (–)	0.00 (–)	0.413
Remifentanil (μg)	1465 (1106)	1885 (1268)	<0.001
Duration of surgery (min)	177 (134)	144 (127)	0.012
Preoperative MME daily usage	0 (0.0)	30 (33)	<0.001
Opioid dosage PCA MME	126 (148)	207 (187)	<0.001
Duration of PCA use (days)	4 (3)	5 (4)	0.005
MME at discharge per day	9 (23)	63 (59)	<0.001
**Surgical specialty *n* (%)**			
Orthopaedic surgery	132 (64.7)	111 (55.0)	—
General surgery	41 (20.1)	54 (26.7)	—
Otorhinolaryngology surgery	8 (3.9)	10 (4.9)	—
Urological surgery	5 (2.5)	6 (3.0)	—
Neurosurgery	5 (2.5)	6 (3.0)	—
Gynaecology/obstetrics surgery	5 (2.5)	6 (3.0)	—
Thoracic surgery	2 (1.0)	3 (1.5)	—
Hand surgery	2 (1.0)	2 (1.0)	—
Vascular surgery	4 (2.0)	4 (2.0)	—

We found no significant association between the ASA physical status classification, length of surgery, the intraoperative remifentanil dosage, and the postsurgical opioid dosage nor the opioid dosage at discharge (all *P*>0.1).

### Factors associated with opioid dose at hospital discharge

The MME at discharge is described by the following formula, which we derived from analysis of our data with linear regression to better visualise the influence of statistically significant variables described below and in Table 2:

MMEatdischarge=0.099+15.435∗IsExperienced+1.144∗MME1+0.909∗Durationofuse+0.043∗OpioiddosagePCAMME

**Table 2:** Overview of variables analysed across pre-, peri-, and postoperative phases, highlighting opioid experience as a consistent influencing factor throughout all stages. Values represent adjusted coefficients from multivariable regression models, with 95% confidence intervals in parentheses. Bolded results indicate statistically significant associations (*P*<0.05). ‘—’ Indicates that the variable was not included or not statistically significant in the respective model. The age × female interaction reflects a sex-specific effect of age on PCA opioid use. Remifentanil refers to intraoperative dosage in μ. MME refers to oral morphine milligram equivalent. PCA, patient-controlled analgesia.

Variable	Intraop. remifentanil (μ)	Postop. PCA MME day^−1^	MME at discharge per day
Age (per year)	**−18.6 (−24.9 to −12.3),*****P*****<0.001**	**−1.80 (−2.98 to −0.61),*****P*****=0.003**	—
Female (*vs* male)	**−403.1 (−627.6 to −178.6),*****P*****<0.001**	**−165.5 (−269.7 to −61.3),*****P*****=0.002**	—
Age × female interaction	—	**2.67 (0.79–4.55),*****P*****=0.006**	—
Opioid-experienced (yes)	**390.4 (169.5–611.3),*****P*****=0.001**	**52.0 (13.1–90.8),*****P*****=0.009**	**15.4 (7.4–23.4),*****P*****<0.001**
Preoperative.opioid (MME day^−1^)	—	**0.86 (0.15–1.57),*****P*****=0.017**	**1.14 (1.00–1.29),*****P*****<0.001**
PCA duration (days)	—	—	0.91 (−0.16 to 1.97), *P*=0.094
PCA opioid dosage (MME)	—	—	**0.043 (0.021–0.066),*****P*****<0.001**

With prior opioid experience (Is Experienced=1)

0.099—baseline MME at discharge per day

Previous experience with opioids was a significant predictor, with prior opioid experience being associated with an increase of 15.4 postoperative MME daily usage at discharge (95% CI 7.4–23.4, *t*=3.8, *P*<0.001). On average, opioid-experienced patients received 63.5 MME day^−1^ at discharge compared with 9.4 MME day^−1^ in opioid-naive patients, representing a 6.7-fold higher requirement (Table 1). Within the opioid-experienced group, the discharge dose was nearly twice their own preoperative daily usage (63.5 *vs* 30 MME day^−1^).

Preoperative MME daily usage showed a strong positive association, with each additional unit of preoperative MME leading to an increase of 1.1 postoperative MME day^−1^ at discharge (95% CI 1.0–1.3, *t*=15.6, *P*<0.001). Duration of PCA use in the postsurgical acute pain phase tended to have a positive association with MME at hospital discharge, without reaching statistical significance. Each additional day of PCA use in the postsurgical phase led to an 0.9 MME day^−1^ increase at hospital discharge (95% CI −0.2 to 2.0, *t*=1.7, *P*=0.094).

**Fig 2:**
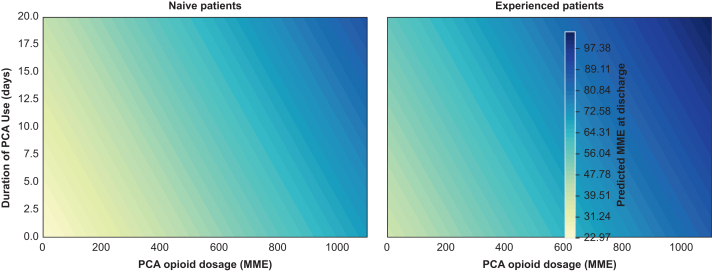
Contour plot showing the combined influence of PCA opioid dosage and duration on postoperative opioid dose at discharge, integrating both model predictions and actual patient data. This visualisation highlights how these factors jointly affect opioid requirements, illustrating the model’s applicability to clinical practice. MME, morphine milligram equivalents; PCA, patient-controlled analgesia.

Opioid dosage via intravenous PCA in the acute postsurgical phase was positively correlated with MME at hospital discharge, with each additional MME of i.v. PCA dosage in the acute postoperative pain phase corresponding to an increase of 0.04 MME at hospital discharge (95% CI 0.02–0.07, *t*=3.8, *P*<0.001). Figure 2 illustrates the combined influence of postsurgical PCA opioid dosage and duration of use on opioid dose (MME) at hospital discharge in both subgroups.

Table 2 provides a structured overview of the significance of key variables across the pre-, peri-, and postoperative phases.

### Factors associated with intraoperative remifentanil dose

The cumulative intraoperative remifentanil dosage was significantly influenced by patient age, sex, and prior opioid experience (Table 2). Age and female sex were negative predictive factors for the needed remifentanil dosage, with each additional year of age 18.6 μ less (95% CI −24.9 to −12.3 μ, *t*=−5.85, *P*<0.001) being needed. Female sex was associated with a decrease of 403.1 μ of cumulative remifentanil dosage (95% CI −627.6 to −178.6 μ, *t*=−3.5, *P*<0.001).

**Fig 3:**
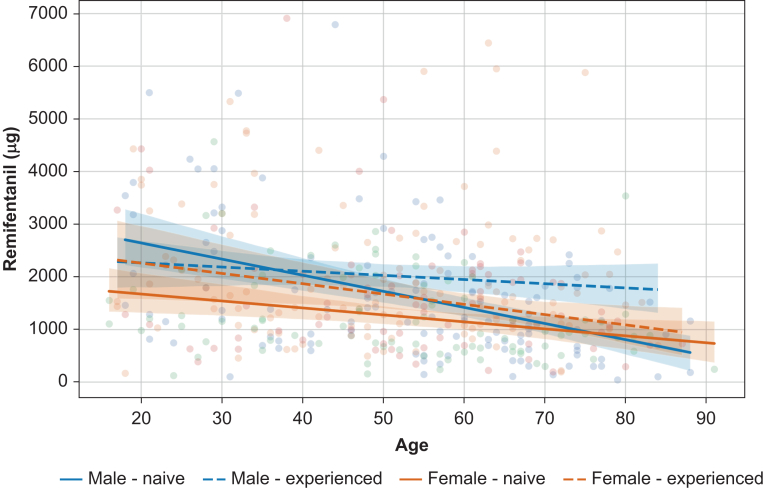
Linear regression lines with 95% confidence intervals are shown. It illustrates the difference of the cumulative intraoperative remifentanil dosage of the analysed patients in the two subgroups. Individual dots represent actual patient-level data.

Patients with prior opioid experience required an additional 390.4 μ of remifentanil (95% CI 169.5–611.3 μ, *t*=3.5, *P*=0.001) during surgery compared with those without such experience. The results are illustrated in Figure 3.

### Factors influencing opioid dosage during the acute postoperative phase (patient-controlled analgesia use)

In this cohort, the PCA pump opioid dosage (MME) in the acute postsurgical pain phase was influenced by four different variables: age, sex, the opioid-naive *vs* opioid-experienced status, and the preoperative opioid dosage (Table 2).

**Fig 4:**
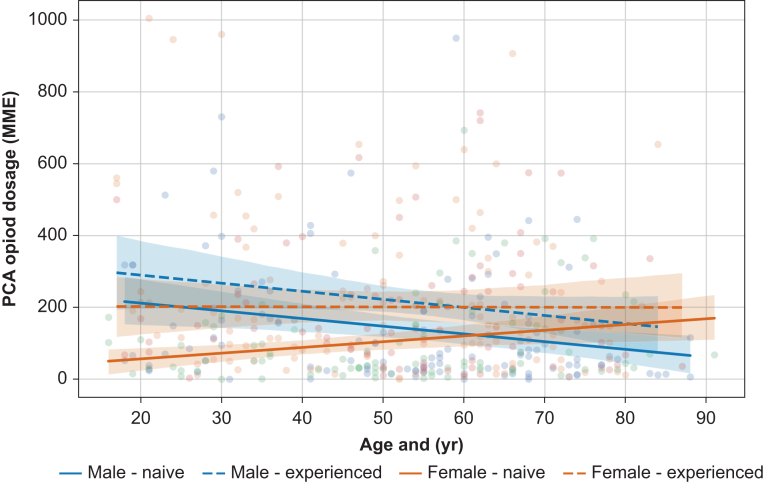
Linear regression of PCA cumulative opioid dosage in MME stratified by age, sex, and opioid status. Lines show regression estimates with 95% confidence intervals; dots represent individual patients. Note the increasing need of MME in the female sex with increasing age and the decreasing need in older men. MME, morphine milligram equivalents; PCA, patient-controlled analgesia.

Overall, age and female gender as single co-variables were found to have a significant negative association with i.v. PCA opioid dosage in the acute postoperative phase, with each additional year of age leading to a decrease of 1.8 MME day^−1^ (95% CI −3.0 to −0.6 MME day^−1^, *t*=−3.0, *P*=0.003) and being female to a decrease of 165.5 MME day^−1^ in PCA opioid dosage in the acute pain phase after the surgery (95% CI −269.7 to −61.3 MME day^−1^, *t*=−3.1, *P*=0.002). Figure 4 illustrates the acute postsurgical phase PCA cumulative opioid dosage in MME stratified by age, gender, and opioid status. This figure highlights that age and sex were also a combined variable, with each additional year of age resulting in an increase of 2.7 MME day^−1^ for female patients compared with male patients of the same age (95% CI 0.8–4.6 MME day^−1^, *t*=2.8, *P*=0.006).

Patients with prior opioid experience required an additional 52.0 MME day^−1^ in the postsurgical phase (95% CI 13.1–90.8 MME day^−1^, *t*=2.6, *P*=0.009) compared with those without such experience. For each additional unit of preoperative MME, the acute postoperative phase i.v. PCA opioid dosage increased by 0.9 MME day^−1^ (95% CI 0.2–1.6 MME day^−1^, *t*=2.4, *P*=0.017).

## Discussion

The primary aim of this study was to compare opioid requirements at hospital discharge between opioid-naive and opioid-experienced patients undergoing surgery with postoperative PCA. On average, opioid-experienced patients required 6.7 times more opioids at discharge than opioid-naive patients (63.5 *vs* 9.4 MME day^−1^; *P*<0.001, Table 1). Within the opioid-experienced group, the discharge dose was almost twice their own preoperative daily usage (63.5 *vs* 30 MME day^−1^), indicating a substantial escalation in opioid dosage at discharge.

These results align with Hofer and colleagues^15^ who demonstrated that long-term postoperative opioid use is predominantly driven by chronic pain unrelated to surgery, with only a limited contribution from chronic postsurgical pain. Our findings extend this by quantifying the magnitude of the difference and confirming that preoperative opioid dose itself was a strong continuous predictor, with each additional MME day^−1^ before surgery associated with a 1.1 MME day^−1^ increase at discharge (95% CI 1.0–1.3 MME day^−1^, *P*<0.001), after adjusting for perioperative factors. This association was further reinforced by the positive link between cumulative PCA use in the acute postoperative phase and discharge MME, underscoring how early postoperative opioid needs can influence prescribing patterns at discharge. Patients with prior opioid experience required an additional 52.0 MME day^−1^ in the acute postsurgical phase compared with opioid-naive patients (95% CI 13.1–90.8 MME day^−1^, *P*=0.009), likely contributing to their elevated discharge doses.

Furthermore, these findings also expand upon earlier work showing preoperative opioid exposure as one of the strongest predictors of postoperative opioid consumption and prescribing. Buckarma and colleagues^16^ similarly reported that opioid-experienced patients required higher discharge dosages and had more frequent refills after surgery.

Our results add novel quantitative insight by providing a prediction model that incorporates preoperative MME, duration of use, and PCA MME in the postoperative acute pain phase, offering a potentially practical tool for tailoring discharge prescriptions. Whereas most previous studies have described the association qualitatively or for specific surgical populations, we quantify it across a heterogeneous cohort, which could improve individualised opioid stewardship.

Clinically, these findings underscore the importance of systematically documenting preoperative opioid status and dosage during the pre-anaesthesia assessment. Integrating such models (e.g. via our prediction equation) into prescribing practice could help ensure adequate pain control while reducing excessive opioid supply—both of which are critical for lowering the risk of persistent postsurgical pain and long-term dependence.^17^^,^^18^

As a secondary aim in this study, we evaluated known factors for higher intraoperative remifentanil requirements—such as increased age, lean body mass, individual pharmacodynamic variability, and procedural factors including surgical invasiveness—and their effect on postsurgical opioid needs via PCA (MME in the acute postoperative phase) and with opioid dosage at discharge.^19^^,^^20^ In addition to preexisting knowledge, this study highlights the importance of previous opioid status/dosage and its effects on the administered opioids given during and after surgery, as the significance of these variables has not been thoroughly researched.

Our model for the intraoperative need for remifentanil highlights that opioid status, age, and sex are important variables for the cumulative dosage intraoperatively. Sex and age displayed a negative association, each additional year of age 18.6 μg less (95% CI −24.9 to −12.3 μg, *P*<0.001) being needed and female sex with a decrease of 403.1 μg of cumulative remifentanil (95% CI −627.6 to −178.6 μg, *P*<0.001). In contrast to the results of Minto and colleagues^20^ who showed no influence of sex on any pharmacokinetic or pharmacodynamic parameter, we found a strong association. Age has previously been shown to influence intraoperative remifentanil dosage.^21^

In 2017, Motamed and colleagues demonstrated that the intraoperative remifentanil requirements were significantly higher for opioid-experienced cancer patients.^22^ Based on our data, however, this appears to be a general trend across different patient groups, not limited to cancer patients. In our cohort, opioid-experienced patients required an additional 390.4 μg of remifentanil (95% CI 169.5–611.3 μg, *P*=0.001) during surgery compared with those without such experience.

We did not find a statistically significant correlation between the intraoperative remifentanil dosage and the cumulative postsurgical opioid PCA dosage in this cohort (*P*=0.173), unlike Chung and colleagues,^10^ questioning the association between perioperative remifentanil dosage and postoperative hyperalgesia. However, this issue was not a primary outcome in our study and the analysis may have been underpowered, which could explain the differing results.

We analysed co-variables that influence the need for i.v. PCA, a topic that has not been thoroughly studied. Interestingly, we found that in male patients, increased age was associated with lower i.v. PCA opioid dosage, whereas in female patients an increase in age led to a need for higher doses of i.v. PCA opioids. Analysed individually, age and female sex remained negative predictive factors. This phenomenon was not specific to a single subgroup of operations, but for all analysed surgeries. There are a couple of hypotheses about how sex differences in pain perception appear to occur across the lifespan and have been linked to differences in central sensitisation. A likely contributor to sex differences in pain perception could be the link between sex hormones and opioid.^23^^,^^24^

However, at present, a clear physiological mechanism explaining the sex differences in pain across the lifespan has not been found. Further studies are needed to explore whether this interaction reflects true biological differences or is confounded by sociocultural or healthcare behaviour patterns in how pain is reported or treated.

Our retrospective single center study has limitations which include but are not limited to selection bias, information bias (missing or incorrect data), and lack of generalizability. Major trauma patients and patients who received neuraxial anaesthesia, regional anaesthesia, or peripheral nerve blocks were excluded to minimise confounding, which may restrict the applicability of our results to broader surgical populations receiving multimodal analgesia. It is important to note that NSAIDs and other non-opioid analgesics were not assessed in this study, and their influence on pain management and opioid use warrants further investigation.

We report that opioid status and cumulative intravenous PCA dosage in the acute postsurgical phase were strong predictive factors for opioid dosage at discharge. Each additional preoperative MME daily usage led to an increase of 1.1 postoperative MME day^−1^ at discharge (95% CI 1.0 to 1.3 postoperative MME day^−1^, *P*<0.001).

For each additional unit of preoperative MME, the PCA opioid dosage after operation increased by 0.9 MME day^−1^ (95% CI 0.2–1.6 MME day^−1^, *P*=0.017), which was statistically relevant to the opioid dosage at discharge. Importantly, opioid-experienced patients required an additional 52.0 MME day^−1^ via PCA in the acute postsurgical phase and greater intraoperative remifentanil doses. These findings underscore the importance of knowing opioid status and dosage on admission. Incorporating this information into preoperative planning could support individualised analgesic strategies, such as our proposed discharge MME prediction formula, to optimise prescribing and reduce unnecessary opioid exposure.

## Conclusions

Preoperative opioid experience strongly predicted postoperative opioid requirements and discharge prescribing. For opioid-experienced patients, who may require substantially higher intraoperative and postoperative opioid doses, perioperative pain management should not rely solely on opioid escalation. Instead, a multimodal approach using non-opioid analgesics (e.g. NSAIDs, acetaminophen, ketamine, gabapentinoids) and regional techniques where feasible may improve pain control, minimise opioid-related side-effects, and reduce tolerance development.^25^ Such strategies are critical for lowering the risk of persistent postsurgical pain and long-term opioid dependence. Adequate pain control in the immediate postoperative period supports earlier mobilisation, improves functional recovery, and reduces complications, while preventing undertreatment at discharge—particularly in opioid-experienced patients—may help avoid the transition to chronic pain states.^26^, ^27^, ^28^ Optimising discharge opioid dosing based on preoperative opioid status balances the risks of overprescribing with preventing undertreatment, ultimately improving both short- and long-term patient outcomes. Our findings also suggest potential sex- and age-related differences in postoperative opioid requirements, indicating that demographic factors may further refine individualised pain management strategies and warrant targeted future research, although these findings were exploratory and not the primary focus of this study.

## Authors’ contributions

Conceptualised and drafted the manuscript, performed the primary research and writing: EL

Provided general support for the project, reviewed the manuscript: UP

Provided critical supervision of the study design, reviewed, and edited the manuscript: AKH

## Declarations of interest

The authors declare that they have no conflicts of interest.
